# Intra-articular injections in sport-active patients with degenerative cartilage lesions or osteoarthritis of the knee: a systematic review

**DOI:** 10.1186/s40634-023-00674-0

**Published:** 2023-11-08

**Authors:** Luca De Marziani, Alessandro Sangiorgio, Alessandro Bensa, Angelo Boffa, Luca Andriolo, Giuseppe Filardo

**Affiliations:** 1 Clinica Ortopedica e Traumatologica 2, IRCCS Istituto Ortopedico Rizzoli, Bologna, Italy; 2 Service of Orthopaedics and Traumatology, Department of Surgery, EOC, Lugano, Switzerland; 3 Applied and Translational Research (ATR) Center, IRCCS Istituto Ortopedico Rizzoli, Bologna, Italy; 4 Faculty of Biomedical Sciences, Università della Svizzera Italiana, Lugano, Switzerland

**Keywords:** Sport, Athletes, Cartilage, Osteoarthritis, Knee, Injective, Hyaluronic Acid, HA, Platelet-rich Plasma, PRP

## Abstract

**Purpose:**

The aim of this systematic review was to analyse the available clinical evidence on intra-articular knee injections for the treatment of degenerative cartilage lesions and osteoarthritis (OA) in sport-active patients.

**Methods:**

A literature search was performed in July 2023 according to the PRISMA guidelines on three electronic databases (PubMed, Cochrane, Web of Science). Studies addressing intra-articular injections for degenerative knee cartilage lesions or knee OA in sport-active patients were included. The Downs and Black’s “checklist for measuring quality” was used to evaluate risk of bias and quality of the included studies.

**Results:**

Only 10 clinical studies for a total of 296 sport-active patients were included, with a publication trend increasing over time. The studies were 9 case series and 1 RCT; 7 studies focused on hyaluronic acid (HA), 2 studies focused on platelet-rich plasma (PRP), while 1 study compared HA and PRP. Overall, safety and positive clinical findings were for both HA and PRP, although not always with satisfactory results in terms of return to sport. The Downs and Black evaluation showed an overall poor quality of the included studies, with an average score of 21.1 points (range 19–25).

**Conclusions:**

The available clinical evidence is still limited, with only a few studies published and an overall low-quality of evidence, suggesting a potential role of HA and PRP injections to treat these patients. However, further high-level trials are needed to confirm the real benefits of these treatments for the management of sport-active patients affected by degenerative cartilage lesions or OA of the knee.

## Introduction

Degenerative cartilage lesions and osteoarthritis (OA) are commonly observed in the sport-active population [31, 55]. They represent one of the most common causes of knee pain and performance deterioration in athletes, with studies showing a higher incidence of knee OA in athletes compared to the general population [1, 22, 40, 45]. This is ascribable to the continuous cartilage solicitation and frequent overuse injuries during physical activity, leading to its premature degeneration, joint inflammation, and ultimately favouring the early development of OA [15, 16, 30, 45]. These patients can experience symptoms ranging from knee pain and loss of function, which negatively impact their sport activity, resulting in reduced performance and even early retirement from sport [55]. First-line treatment is non-surgical, relying on several conservative strategies ranging from oral medications to physiotherapy [3, 13, 34, 50]. However, these treatments often result in suboptimal recovery [22, 36]. Other surgical procedures addressing the articular surface, the alignment, as well as ligament and menisci, are not always indicated [55], and total knee arthroplasty represents an end stage solution for older patients affected by OA, but it does not represent a suitable option in younger patients, due to their high expectations and functional demands [5, 42].

Intra-articular injective treatments emerged in recent years as an alternative minimally invasive option for the management of degenerative cartilage lesions and OA in sport-active patients [29]. These therapies have been proposed to provide a clinical benefit and delay more sacrificing procedures, avoiding the impact and risks of surgical treatments in these active patients. Numerous pre-clinical studies demonstrated that intra-articular injective treatments could provide disease-modifying effects in animal OA models, attenuating cartilage damage progression and reducing synovial inflammation [11, 12, 44]. Moreover, increasing clinical evidence documented the clinical benefits offered by injective treatments in the general population suffering from knee OA [17, 21, 26]. Nevertheless, sport-active patients do not match the characteristics and the needs of the general population, representing a unique category of patients with challenging functional requirement that should be appropriately addressed. A recent survey performed in FIFA Medical Centers of Excellence focusing on the preferred management strategies of soccer players affected by knee cartilage injuries, including degenerative lesions and OA, reported that injective treatments represent one of the most used approaches to address these patients [35]. However, despite an increasing use of knee injections in the clinical practice to address sport-active patients, no consensus on the best injective strategy has been reached and the efficacy itself is controversial, leaving the management of this specific population a debated topic.

The aim of this systematic review was to analyse the available clinical evidence on intra-articular knee injections for the treatment of degenerative cartilage lesions and OA in sport-active patients.

## Materials and methods

### Search strategy and article selection

A literature search was performed on July 06, 2023, according to the Preferred Reporting Items for Systematic Reviews and Meta-Analyses (PRISMA) guidelines, on three electronic databases (PubMed, Cochrane, and Web of Science). The following research terms were used: “(sport*) AND (knee) AND (inject* OR intra-articular OR intra articular OR infiltration) AND (cartilage OR chondral OR osteoarthritis OR OA)”. Inclusion criteria were studies addressing intra-articular injections for degenerative knee cartilage lesions or knee OA in professional or amateur sport-active patients. Only studies written in English were included. Case reports or case series describing less than 5 cases and articles in languages other than English were excluded. Pre-clinical, ex vivo studies, congress abstracts, and review articles were also excluded. Reference lists from the selected papers and from the systematic reviews found with the first and second screening were also considered, and all selected studies were included in the qualitative data synthesis.

### Data extraction, assessment of risk of bias and quality of evidence

**Fig. 1:**
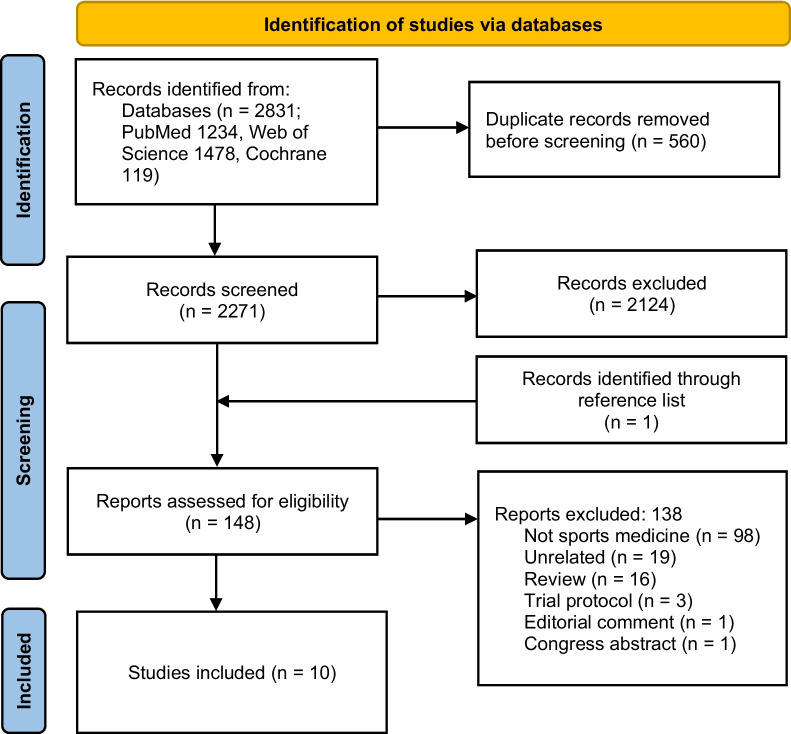
Preferred Reporting Items for Systematic Reviews and Meta-Analyses (PRISMA) flowchart

Two independent reviewers (A.S. and A.Be.) screened all the articles on the title and abstract to assess whether they met the inclusion criteria. After the first screening, the articles that met the inclusion criteria were evaluated for full-text eligibility and were excluded if they did not meet the inclusion criteria (Fig. 1). In case of disagreement between the two reviewers, a third reviewer (L.D.M.) was consulted to reach a consensus. Data were independently extracted on a data extraction form using Excel (Microsoft). The following data were extracted: author, year of publication, number of patients, gender, mean age, type of sport, injected product, safety, and clinical outcomes.

The Downs and Black’s “checklist for measuring quality” was used to evaluate risk of bias and quality of the included studies [18]. This checklist contains 27 ‘yes’-or-’no’ questions across five sections, providing a numeric value up to 32 points. The five sections include questions about the study overall quality (10 items), the ability to generalize findings (3 items), the study bias (7 items), the confounding and selection bias (6 items), and the power of the study (1 item). Assessment of risk of bias and quality of evidence was completed independently for all outcomes by two authors (A.S. and A.Be.) and a third author (L.D.M.) solved any possible discrepancy.

## Results

### Article selection and characteristics

**Fig. 2:**
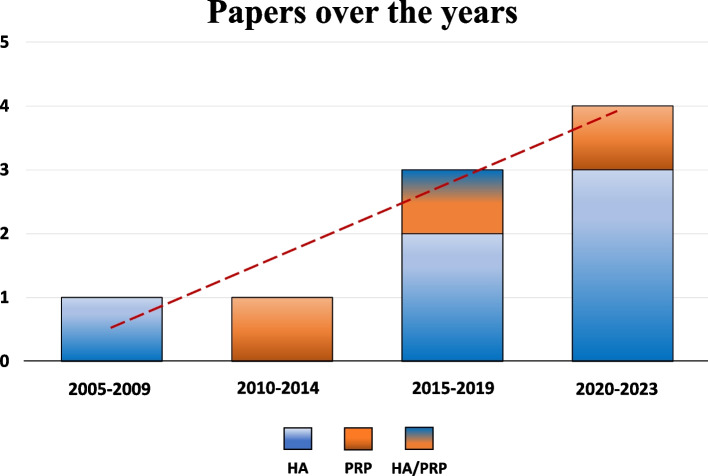
Number of articles published over time on injective therapies in sport-active patients

After duplicates were removed, the initial search identified 2,271 records, whose abstracts were screened and selected according to the inclusion/exclusion criteria for a total of 148 articles assessed for eligibility. One of these articles was identified through the reference lists. After full-text evaluation, 98 studies were excluded as they did not evaluate sport-active patients, 19 were unrelated articles not concerning intra-articular injective treatments of the knee, 16 were reviews, 3 were trial protocols, 1 was an editorial comment, and 1 was a congress abstract. Thus, a total of 10 clinical studies focusing on intra-articular injective treatments for the management of degenerative knee cartilage lesions or knee OA in sport-active patients were included in this systematic review. Since the first report in 2008, the publication trend increased over time, with the 50% of the articles being published from 2019 (Fig. 2).

Among the included studies, the evaluation by study type showed 8 prospective case series, 1 randomized controlled trial (RCT), and 1 retrospective case series. Two injective products were investigated: 7 studies focused on intra-articular injections of hyaluronic acid (HA), 2 studies focused on intra-articular injections of platelet-rich plasma (PRP), while 1 study analysed the comparison between HA and PRP. A total of 296 sport-active patients (237 men and 59 women) treated with intra-articular injections were evaluated: 177 were treated with HA (140 men and 37 women) and 119 with PRP injections (97 men and 22 women). Out of the 10 included studies, 8 studies reported the type of sport played by the patients, of which 5 focused on football players (134 patients), while the other 3 focused on different sports including skiing, motocross, basketball, volleyball, jogging, tennis, bicycling, walking, trekking, golf, jai alai, or couple dance. The other two studies did not specify the type of sport played. Among the included studies, all studies specified the presence of degenerative cartilage lesions or OA as inclusion criteria. The severity of OA was defined in all studies (Kellgren-Lawrence < 3), except for one. The trial duration varied from 6 to 24 months of follow-up, with an average of 9.4 months. The visual analogue scale (VAS) for pain (7 articles), the International Knee Documentation Committee (IKDC, 6 articles), and the Knee injury, and Osteoarthritis Outcome Score (KOOS, 5 articles) were the most used scores. Other scores, such as the Western Ontario and McMaster Universities Osteoarthritis Index (WOMAC), the Tegner score, the Lysholm knee scoring scale, and the EuroQol Visual Analog Scale (EQ-VAS) were used in less than 5 articles. Among the included articles, two studies declared no funding, two studies on HA received sponsor founding, while the remaining six articles did not report such information. The number of injections varied between studies from 1 to 3. Two studies used a single injection of HA, 4 studies (3 on HA and 1 on PRP) evaluated the results of an injection cycle of 2 injections, while 4 studies, including the only RCT, evaluated the results of an injection cycle of 3 injections (2 HA, 1 PRP, and 1 HA vs PRP). Further characteristics of the included studies and the injected products are reported in Table 1 and in the following paragraphs.

**Table 1:** Characteristics of the included studies

**Authors**	**Inclusion criteria**	**Study design**	**N° Of patients (M/F)**	**Age** **(sd)** **[range]**	**Practiced sport**	**Injected product**	**Injective protocol** Volume N. Injection Interval	**Scores**	**Follow-up**	**Main findings**
Zietz et al. 2008 [58]	Outerbridge grade III or IV Previous knee arthroscopy with residual pain/no desired return to sport	Prospective case series	15 (10/5)	49.5 (/) [34–59]	4 professional jai alai, 11 recreational basketball, golf, skiing, jogging	Hylan G-F 20 (Genzyme, Cambridge, MA) 6000 kDA	/ 3 injections 1-week interval	IKDC WOMAC Activity level	3–6 m	Improved activity levels and sports participation at 3 m follow-up. No difference in IKDC and WOMAC
Gobbi et al 2012 [23]	Age 30–60 y Kellgren-Lawrence 1–3 Severe pain > 3 m Stable knees, normal knee alignment, normal patellofemoral tracking	Prospective case series	50 (31/19)	47.7 (/) [32–60]	Football (14%), skiing (14%), motocross (12%), basketball/volleyball (12%), jogging (10%), and others (tennis, bicycling, walking, trekking, etc.) but not at a professional level	PRP Autologous Fresh Code: XX-XX-00	4 mL 2 injections 1-month interval	VAS IKDC Tegner KOOS	6–12 m	Encouraging preliminary clinical results was found in active patients with knee OA
Papalia et al. 2016 [41]	Degenerative cartilage lesions Symptomatic unilateral knee pain Unresponsive to conservative therapies No previous surgery No major axial deformities	RCT	47 (47/0)	37.2 (/) [34–39]	Professional football players at the end of their career	SINOVIAL HL (IBSA, Lodi) 1100–1400 kDA and 80–100 kDA	64 mg/2 mL 3 injections /	IKDC KOOS VAS	3, 6, 12 m	Both treatments showed to be effective in relieving patients’ symptoms. HA showed higher effectiveness compared to PRP, although its superiority was limited by time
						PRP Autologous Fresh Code: XX-XX-00	5.5 mL 3 injections /			
Tamburrino et al. 2016 [51]	Femoropatellar/femorotibial chondropaty ICRS Grade ≤ 3a	Prospective case series	30 (30/0)	30.7 (/) [17–39]	Professional football players	HYADD4-G (Fidia Farmaceutici) 500–730 kDA	24 mg/3 mL 2 injections 1-week interval	VAS KOOS	1, 3, 6 m	HA is an effective therapeutic option for patients with knee degenerative or traumatic chondropathy
Wu et al. 2017 [56]	Kellgren-Lawrence 1–2	Prospective case series	18 (18/0)	/ (/) [25–38]	Football	ArtiAid (Maxigen Biotech inc. Taiwam) 600–1200 kDA	2.5 mL 3 injections 1-week interval	IKDC WOMAC	1–4 w	Significantly improved IKDC and WOMAC scores
Migliore et al. 2019 [38]	Age ≥ 18 y Kellgren-Lawrence 1–2	Retrospective case series	17 (16/1)	39.8 (± 11.8) [/]	Football players (3 professional, 14 non-professional)	SINOVIAL HL (IBSA, Lodi) 800–1200 kDA	16 mg/2 mL 2 injections 2-week interval	Lequesne index score VAS	1, 2 d 2, 4 w 3, 6 m	Stable improvement of symptoms, rapid restart of sports activity
Altamura et al. 2020 [1]	Age ≤ 50 y Unilateral knee pain for at least 4 m Kellgren-Lawrence 0–3 Any level of sport	Prospective case series	47 (44/3)	41.1 (± 7.1) [/]	NR	PRP Autologous Cryopreserved Code: XX-X1-11	5 mL 3 injections 1-week interval	IKDC subjective EQ-VAS Tegner	2, 6, 12, 24 m	Pain and function improvement up to 24 months. However, only half of the patients can achieve the same sport level as before
Bernetti et al. 2020 [9]	Age 30–70 y Kellgren-Lawrence 2–3 Amateur athletes	Prospective case series	30 (22/8)	60.4 (/) [/]	15 runners, 4 football, 5 tennis, 6 couple dance	HYADD4-G (Fidia Farmaceutici) 500–730 kDA	24 mg/3 mL 2 injections 2-week interval	VAS KOOS WOMAC	1, 3, 6 m	Intra-articular HA injections represent a safe method in athlete management with low-moderate knee OA
Bernetti et al. 2021 [8]	Age 18–65 y Kellgren-Lawrence 1–3 Professional or regular sport player No pain medications 48 h before visit	Prospective case series	31 (8/23)	49 (/) [/]	NR	HYADD4-G (Fidia Farmaceutici) 500–730 kDA	32 mg/4 ml 1 injection /	KOOS WOMAC VAS	1, 3, 6, 12 m	A single HA intra-articular injection seems to provide a rapid, lasting, and safe response in regular sports players affected by knee OA
Perticarini et al. 2021 [43]	Age ≥ 18 y Knee chondropathy at MRI No inflammatory signs of the joint No ligament/meniscal pathologies No surgery	Prospective case series	12 (12/0)	NR	Professional football players	HYADD4-G (Fidia Farmaceutici) 500–730 kDA	32 mg/4 mL 2 injections 19/20-week interval	0–4 VAS IKDC Lysholm	40 w	A single HA injection, repeated after 19–20 weeks, may be a viable option to improve symptoms and function in professional players

*D* days, *F* Female, *HA* Hyaluronic acid, *ICRS* International Cartilage Repair Society, *IKDC* International Knee Documentation Committee Subjective Knee Form, *y* Years, *kDA* kilo daltons, *KOOS* Knee injury and Osteoarthritis Outcome Score, *M* Male, *m* months, *NR* not reported, *PRP* Platelet-rich plasma, *sd* Standard Deviation, *VAS* Visual analogic scale, *w* weeks, *WOMAC* Western Ontario and McMaster University Osteoarthritis index. PRP code according to Kon et al. [28]

The evaluation with the Downs and Black checklist showed an overall poor quality of the included studies, with an average score of 21.1 points (range 19–25) as reported in Table 2.

**Table 2:** Methodological quality of the included studies with evaluation

Articles	Reporting	External validity	Internal validity bias	Internal validity confounding	Power	Total
**Zietz et al. 2008** [58]	9	3	5	2	0	**19**
**Gobbi et al. 2012** [23]	10	3	5	2	2	**22**
**Papalia et al. 2016** [41]	9	3	7	6	0	**25**
**Tamburrino et al. 2016** [51]	9	3	5	3	0	**20**
**Wu et al. 2017** [56]	9	3	5	3	0	**20**
**Migliore et al. 2019** [38]	10	3	5	3	0	**21**
**Altamura et al. 2020** [1]	10	3	5	3	0	**21**
**Bernetti et al. 2020** [9]	8	3	5	3	0	**19**
**Bernetti et al. 2021** [8]	11	3	5	2	2	**23**
**Perticarini et al. 2021** [43]	11	3	5	2	0	**21**

### HA injections

Seven studies specifically focused on intra-articular HA injections in sport-active patients. These studies evaluated different HA types: 5 studies evaluated low molecular weight HA, 1 study evaluated a medium molecular weight HA, and 1 study evaluated a high molecular weight HA. Among the different products, the HYADD4-G (Fidia Farmaceuditici, Abano Terme, Italy, 500–730 kDA) was the most studied (4 studies), while the ArtiAid (Maxigen Biotech Inc. Taiwan, 600–1200 kDA), the SINOVIAL HL (IBSA Pharma inc., Lugano, Switzerland, 800–1200 kDA), and the Hylan G-F 20 (Genzyme, Cambridge, MA, 6000 kDA) were evaluated in one study. More in detail:

- Two studies evaluated the clinical results of two injections of HYADD4-G (24 mg/3 ml), an HA with a mobile reticulum. Both studies reported positive results in terms of pain relief after HA injections, with a significant improvement in the VAS scale documented up to 6 months of follow-up, despite controversial results in the other outcomes. In fact, Tamburrino et al. [51] highlighted a significant improvement in all KOOS subscales at 6 months in 30 male professional soccer players (mean 30.7 years old) treated with two injections (one-week interval), with all patients returning to play soccer within 6 months relatively pain free. On the other hand, Bernetti et al. [9] documented a significant improvement only for KOOS Pain and Quality subscales and no improvement for the WOMAC score at 6 months of follow-up in 30 amateur athletes (22 men, 8 women, mean 60.4 years old) treated with two injections (two-week interval). Other two studies evaluated a different formulation of HYADD4-G (32 mg/4 ml). In the study of Bernetti et al., a single intra-articular injection of this HA provided a rapid and lasting clinical response in 31 regular sports players affected by knee OA (8 men, 23 women, median 49 years old), with a significant improvement documented in the VAS, KOOS, and WOMAC scales at all follow-ups up to 12 months [8]. In the study of Perticarini et al., two injections in 12 male professional soccer players (age not reported) at the beginning and in the middle of the sport season led to a significant improvement in the VAS, IKDC, and Lyshom scales at the end of the season compared to the beginning [43].
- The study of Wu et al. evaluated the clinical effect of three intra-articular injections (one-week interval) of the low molecular weight ArtiAid (2.5 ml) in 18 male football players (age 25–38 years old) affected by Kellgren-Lawrence grade 1–2 knee OA, reporting a significant improvement in WOMAC and IKDC scores after 1–4 weeks from the treatment [56]. Moreover, players underwent an immunochromatographic urine strip for the analysis of cartilage oligomeric matrix protein (COMP), a degradation cartilage marker, revealing an improvement in 70% of the patients.
- The study of Migliore et al. assessed the clinical results of two injections (two-week interval) of a medium molecular weight HA (SINOVIAL HL, 16 mg/2 ml) in 17 football players (16 men, 1 woman, mean 40 years old) affected by Kellgren-Lawrence grade 1–2 knee OA, showing a rapid return to sport activity and a significant improvement of VAS and Lequesne index at 1-3-6 months [38].
- The study of Zietz et al. analyzed the effect of a high molecular weight HA (Hylan G-F 20) in the treatment of 15 patients (10 men, 5 women, mean 49.5 years old) with knee OA who had undergone knee arthroscopy for partial meniscectomy and complained of residual pain [58]. The authors reported that three weekly HA injections after knee arthroscopy increased the patients’ activity level (4 returned to professional level, the others to recreational level), while they were not able to improve the IKDC and WOMAC scores at 3–6 months of follow-up compared to the post-surgery.

Regarding the safety of HA injective treatments for sport-active patients, 6 out of these 7 studies reported on adverse events that occurred “few local side effect”. While one study reported “few local side effect” without specifying the type of side effect and the number of involved patients, the other 5 studies documented mild adverse events in 4/105 patients (3.8%), including self-limiting joint pain and acute local reaction. No severe adverse events were documented. One study did not report any data on the safety of the injective treatment.

### PRP injections

Two studies specifically focused on intra-articular injections of autologous PRP in sport-active patients. In detail:

- The study of Gobbi et al. [23] analysed 50 active patients (31 men, 19 women, mean 47.7 years old) affected by symptomatic knee OA and treated with two intra-articular PRP injections (4 ml), with an injection interval of 1 month, and evaluated up to 12 months of follow-up. The used PRP was autologous, fresh, and without external activation, other product characteristics were not documented. In this study, a significant improvement in subjective IKDC, KOOS, and Tegner scores was reported at 6 and 12 months, with all patients returning to their previous sport activity level. Authors divided patients in two groups based on previous knee surgery, finding no significant difference between the two groups. Moreover, no significant difference was reported between men and women.
- The study of Altamura et al. [1] analysed 47 sport-active patients (44 men, 3 women, mean 41.1 years old) with unilateral symptomatic knee cartilage degeneration or OA treated with 3 weekly intra-articular PRP injections (5 ml) and evaluated up to 24 months of follow-up. The used PRP was autologous, cryopreserved, and with a concentration of platelets and leukocytes of 4.6 and 1.1 higher than their baseline blood value, respectively. PRP was activated adding 10% of calcium chloride. This study demonstrated that these patients can benefit from PRP injections, with pain and function improvement over time. However, results were less satisfactory in terms of return to sport since, while 77% returned to some sport activity, only half of the patients achieved the same sport level as before the onset of symptoms. Moreover, a lower pre-symptoms Tegner score was associated with a higher grade of return to sport.

The safety of PRP injections was documented in both studies: no adverse events were reported during the procedures and at follow-up.

### HA vs PRP injections

Only one RCT investigated the clinical results of injective treatments for sport-active patients affected by cartilage degenerative lesions or OA (Kellgren-Lawrence grade 1–2) [41]. In this study of Papalia et al., 47 male end-career professional soccer players (mean 37.2 years old) were randomized to receive 3 intra-articular injections of HA or PRP. The authors used a hybrid HA (Synovial HL, 3,2%, 64 mg/2 ml) composed of 32 mg of high-molecular weight (1100–1400 kDa) hyaluronan and 32 mg of low-molecular weight (80–100 kDA) hyaluronan. The used PRP was autologous, while the interval between injections, the concentration of platelets and leukocytes of the injected PRP, and the PRP activation method were not reported. Both injective treatments proved to be effective in clinical terms of improvement at all follow-ups. The hybrid HA group showed a significant superiority compared to PRP group in terms of VAS, IKDC, and KOOS scores at 3 and 6 months of follow-up, although the intergroups differences decreased gradually until losing significance at 12 months of follow-up. Regarding the safety of the treatments, no adverse events were highlighted following the procedures or in the follow-ups analyzed.

## Discussion

The main finding of this systematic review is that the available clinical evidence on the use of injective treatments for knee degenerative cartilage lesions and OA in sport-active patients is limited, with only few studies published and an overall low-quality level of evidence. This makes it difficult to draw clear conclusions on the real efficacy of the injective approach in this complex clinical setting.

The active sports population suffering from knee cartilage lesions or OA represents a challenge for clinicians due to the presence of compromised knees in relatively young patients who still have high expectations and functional sports requirements [1]. In particular, the possibility of returning to the same activity level is paramount in this type of sport active population when deciding to undergo a specific treatment. An acceptable outcome might be difficult to reach in these patients, and the positive results obtained in the general population could not be directly translated to them with a return to the previous level of sport activity [17, 21]. In this light, poor results in sport-active patients have already been reported for different treatments. For example, a low rate of return to pre-injury sport level has been described in athletes after cartilage surgery at long-term follow-up, despite a marked improvement in all clinical scores analyzed [57]. Similarly, it has been described that sport-active patients who underwent high tibial osteotomy to correct varus knee malalignment can obtain positive results in terms of pain relief, but the return to sport is not always satisfactory [4, 7, 19, 49].

Poor results for sport-active patients have been also suggested for the knee arthroplasty, which represents the end-stage treatment of knee OA. In fact, whereas the joint replacement can offer a satisfying functional recovery in older and less active patients, this type of treatment could not represent the ideal solution for young sport-active patients, due to an increased risk of implant failure and wear [2, 24, 37, 42, 46]. For these reasons, there is a need to find new solutions for sport-active patients affected by degenerative cartilage lesions and OA of the knee in order to avoid or delay the need for joint replacement and at the same time allow them to return to their sport activity level. Among the suggested treatments, intra-articular injections have been proposed as minimally invasive options able to improve the symptoms and possibly favor the return to sport.

This systematic review documented an increasing interest in injective treatments for knee degenerative cartilage lesions and OA in sport-active patients, with 50% of the clinical studies being published in the last 4 years. This is closely related to the increasing interest in injective treatments for the management of knee OA in the general population, together with the positive results in this setting [14, 17, 21]. Nevertheless, the number of specific studies focusing on sport-active patients affected by knee degenerative cartilage lesions and OA is still limited, as well as the number of patients analyzed which is lower than 300. Furthermore, the level of evidence and the quality of the studies currently available in the literature has proven to be poor. To date, there is only one RCT of modest size (less than 50 patients), while the other available studies are prospective or retrospective case series without a control group. Moreover, another important aspect to be taken into account is that only two of the included studies declared that they did not receive funding for the execution of the study, two studies reported receiving funding from a pharmaceutical company, while the other six studies did not provide any information. This could put these studies at risk of bias, considering that it has been suggested that sponsored studies are more likely to report findings that favor the sponsor, underlying the need for independent research efforts to confirm the findings obtained from these studies [10].

In addition to the limited number of available studies, it should be noted that the evidence on injective treatments for sport-active patients is also characterized by high heterogeneity in terms of evaluated patients. The effects of injective treatments were assessed in patients playing different sport activities and at difference level (professional vs amateur level). The type and level of sport played by patients is crucial, as it has been demonstrated how different sports result in different types of joint and cartilage stresses [30, 45, 54]. The overall analysis of patients practicing different sports could be therefore affected by the different functional demands of these patients. This is further complicated by the high heterogeneity in terms of patient age documented in the analyzed series which ranged from a mean of 30 to 60 years old, a key factor that could influence the results both in terms of activity level and joint stress, as well as biological potential and response from the joint environment of young versus old patients [27]. Therefore, future studies should analyze the clinical outcomes standardizing patients practicing the same type of sports and having similar age and functional demands.

A high heterogeneity in this field has been observed also in terms of the injective products used, even though only two types of products were analyzed: HA and PRP. Viscosupplementation demonstrated clinical benefits in sport-active patients up to 12 months of follow-up [8, 43], with one study also reporting molecular benefits at cartilage level evaluated through a urine test [56]. However, the 8 studies reporting on HA injections evaluated 5 different products, with different molecular weight, different volumes, and different injective protocols, hindering the possibility to perform a meta-analysis. Similarly, also the 3 studies reporting on PRP presented heterogeneity in terms of product characteristics and injection protocols, with different PRP production techniques, different injected volumes, different activation methods, and different platelets and leukocyte concentrations. All these differences in product characteristics and injective protocols should be investigated in specific clinical trials to identify the parameters that could optimize a specific injective treatment for the management of sport-active patients.

Future high-level studies should confirm the preliminary positive results documented by the low-level studies currently available in the literature. In fact, only one RCT investigated the role of intra-articular injective treatments in sport-active patients [41]. This trial reported interesting findings, demonstrating that 3 intra-articular injections of a hybrid HA provided better results compared to 3 intra-articular injections of PRP in end-career professional soccer players at 3 and 6 months of follow-up, although the differences between the two groups were not confirmed at 12 months. These results are surprising considering the general literature on knee injections for knee OA patients. In fact, different meta-analyses supported the superiority of PRP injections over HA in the general population, with higher clinical benefits provided by PRP especially at longer follow-up [6, 21, 52]. Future high-level trials should compare these products in the management of sport-active patients, to understand if HA and PRP present different indications, and confirming the most suitable treatment option also by comparing them with the placebo effect.

The placebo effect plays a major role in injective treatments, especially in case of new attractive products [47]. The contribution of the placebo effect in terms of pain relief is very relevant for knee OA injections, being not only statistically but also clinically significant, with clinical benefits perceived up to 6 months of follow-up [47]. The placebo effect is present and significant also in the treatment of sport-active patients [25, 53]. For example, it has been demonstrated that athletes who falsely believed they had been administered anabolic steroids performed better than their baseline or when compared with controls [33]. Similarly, another study reported significant improvements in 3000-m running time when participants self-injected intra-muscle saline, which they believed was a substance similar to recombinant erythropoietin [48]. In this scenario, a significant placebo effect could be present also in the intra-articular injective treatment of sport-active patients. Therefore, double-blind placebo controlled RCTs are needed to confirm the real benefit offered by these treatments in sport-active patients, as only treatments that statistically and clinically outperform the placebo effect should be performed in the clinical practice.

Further studies should also investigate possible factors that could influence the efficacy of the different injective treatments in sport-active patients affected by knee degenerative cartilage lesions or OA. To this regard, an important aspect to consider is that only 20% of the patients were women and only one study evaluated results considering sex [23]. Investigating differences between men and women is a pressing issue in orthopedics. Men and women present numerous physiological and pharmacological differences, and male overrepresentation could influence the understanding of efficacy of a specific injection treatment in women, as already reported for injective treatments in the general population [20, 32]. In this light, there is a need for greater representation of women in studies and the reporting of sex-stratified data in order to understand if there are different risks and different clinical outcomes with injective treatments in sport-active women [20, 39]. This and other aspects of the treated patients and of the products should be investigated to optimize the use of injective treatments in sport-active patients affected by knee degenerative cartilage lesions or OA.

The limitations of this systematic review reflect the limitations of this field. The literature analysis showed that the clinical evidence is very limited and characterized by a low-level of evidence with only one RCT with a small size and without a placebo-controlled arm. Moreover, high heterogeneity was observed in terms of injected products, injective protocols, and evaluated patients. Similarly, the included studies did not always report the exact number of adverse events and used different definitions, hindering the possibility to obtain an accurate adverse event rate. Finally, there are not enough stratified and homogeneous data based on the type of injected product, making it difficult to merge and compare clinical results, thus impairing the possibility to perform a reliable meta-analysis to draw clear conclusions. Future studies should analyze clinical results better stratifying by product and patients’ characteristics according to gender, type of sport practiced, age, and functional demands. High-level studies should confirm the preliminary positive results currently available in the literature by comparing the products used also with placebo. Those and other features of included patients and products should be evaluated to improve the management of sport-active patients with degenerative cartilage lesion of the knee or OA.

Nevertheless, this systematic review offered a comprehensive state-of-the-art picture of the field, underlining overall positive clinical results, although not always optimal in terms of return to sport. However, considering the limitations of the available literature, the increasing use of these treatments in the clinical practice does not appear to be sufficiently supported by the current evidence. Further high-level studies are necessary to better elucidate the real therapeutic potential, the most suitable indications, and the optimal product and approach to use intra-articular injective treatments to address sport-active patients affected by degenerative cartilage lesions or OA of the knee.

## Conclusions

This systematic review documented an increasing interest on knee intra-articular injections for the treatment of sport-active patients affected by knee degenerative cartilage lesions or OA, although the available clinical evidence is still very limited, with only few studies published and an overall low-quality of evidence level. Overall, positive clinical findings have been reported for both HA and PRP, although not always with satisfactory results in terms of return to sport. Further high-level trials are needed to confirm the real benefits of these treatments for the management of sport-active patients affected by degenerative cartilage lesions or OA of the knee.

## Data Availability

Not applicable.
